# The Effect of *Thymus vulgaris* L. Hydrolate Solutions on the Seed Germination, Seedling Length, and Oxidative Stress of Some Cultivated and Weed Species

**DOI:** 10.3390/plants11131782

**Published:** 2022-07-05

**Authors:** Bojan Konstantinović, Milena Popov, Nataša Samardžić, Milica Aćimović, Jovana Šućur Elez, Tijana Stojanović, Marina Crnković, Miloš Rajković

**Affiliations:** 1 Department of Phytomedicine and Environmental Protection, Faculty of Agriculture, University of Novi Sad, 21000 Novi Sad, Serbia; bojan.konstantinovic@polj.edu.rs (B.K.); natasa.samardzic@polj.uns.ac.rs (N.S.); jovana.sucur@polj.uns.ac.rs (J.Š.E.); tijana.stojanovic@polj.edu.rs (T.S.); marina.crnkovic@polj.uns.ac.rs (M.C.); 2 Institute of Field and Vegetable Crops Novi Sad, 21101 Novi Sad, Serbia; milica.acimovic@ifvcns.ns.ac.rs (M.A.); rajkovicmilos@gmail.com (M.R.)

**Keywords:** oxidative stress, germination, inhibition, biopesticide

## Abstract

The aim of this study was to determine the effect of the hydrolates obtained as the by-products of the *Thymus vulgaris* essential oil steam distillation process. The bioassays, which were undertaken in order to determine the effect on germination and initial growth of seedlings of some cultivated and weed species, were performed under controlled conditions with different concentrations of the hydrolates. Seeds of *Glycine max*, *Helianthus annuus*, *Zea mays*, *Triticum aestivum*, *Daucus carota* subsp. *sativus*, *Allium cepa*, *Medicago sativa*, and *Trifolium repens*, and six weed species—*Amaranthus retroflexus*, *Chenopodium album*, *Portulaca oleracea*, *Echinochloa crus-galli*, *Sorghum halepense*, and *Solanum nigrum*—were treated with 10, 20, 50, and 100% *T. vulgaris* hydrolate solution. The obtained results showed that the *T. vulgaris* hydrolate had the least negative effect on the germination of cultivated species, such as soybean, sunflower and maize, whereas clover and alfalfa were the most sensitive. By comparison, all the tested weed species expressed high susceptibility. It can be concluded that the *T. vulgaris* hydrolate has an herbicidal effect, in addition to its potential as a biopesticide in terms of integrated weed management.

## 1. Introduction

The hydrolates or hydrosols are the by-products of the essential oil steam distillation process. They consist of condensate water and small amounts of essential oil compounds, which are mostly soluble in water [1]. Hydrosols have been recently noticed for their biological properties, including antifungal, antibacterial and antioxidant activity [2,3]. Today, hydrolates are widely used in the pharmaceutical, food, and cosmetic industries, and in aromatherapy [4]. Their production is easy and cheap, and they appear to be less toxic to human health than essential oils [2].

*Thymus vulgaris* L. is a perennial flowering aromatic plant that has been used globally for many centuries for medicinal and culinary purposes [5]. Essential oils and lipophilic substances are abundant in this plant [6]. According to [7], *T. vulgaris* extracts are rich in the aromatic compounds thymol, carvacrol, p-cymene, and γ-terpinene. The chemical composition of an essential oil depends on the harvesting time of the plant material, the phenological phase of the plant, the growing area of the plants, and other parameters [8]. Thus, the results of chemical composition studies of *T. vulgaris* essential oil and hydrolates vary. For example, it was determined that the major component of *T. vulgaris* essential oil is thymol (36.1%), whereas, in the case of hydrosol, thymol (98.1%) and carvacrol (1.9%) were dominant [9]. According to [8], the major component of the essential oil obtained before flowering is thymol (46.74%), whereas [10] discovered that *T. vulgaris* essential oil contained 68.1% thymol and 3.5% carvacrol.

Thymol and carvacrol have antioxidant properties [11,12]. In the fields of human and veterinary medicine, and in plant protection, the antibacterial and antifungal activity of thymol and carvacrol [13,14,15], the nematocidal effect of *T. vulgaris* essential oil [16], and the antiviral properties of the extracts [17,18,19], essential oil [20,21,22], and hydrolates [23] have been reported. With regard to its antibacterial properties, *T. vulgaris* hydrolate has practical applications in the food industry for the microbiological safety of fresh-cut tomatoes and cucumbers [24], carrots and apples [25], and lettuce [26].

In recent years, in order to reduce the use of pesticides in the control of diseases, pests, and weeds in agriculture, great efforts have been made to identify new biopesticides that are as effective as synthetic chemicals. Thymol and carvacrol are examples of bioactive compounds that may have the potential to become an integral part of agricultural practice as biopesticides. These compounds are potentially useful in controlling the emission of odors and pathogens in swine waste [27]. Due to its fast degradation rates in tropical soil and water, thymol is considered to have a low environmental risk in terms of tropical crop production [28]. According to [29], thymol and carvacrol showed high inhibition at low concentrations against weed seeds, such as those of red-root amaranth (*Amaranthus retroflexus* L.), wild radish (*Raphanus raphanistrum* L.), and wild mustard (*Sinapis arvensis* L.). In the case of *Sorghum bicolor* L. seeds, carvacrol applied in a concentration of 3 mmol L^−1^ showed higher efficacy than the tested commercial herbicide (2,4-D), leading to the inhibition of the germination rate (~40%) and the germination speed (~56%) [30]. Furthermore, it was concluded that, among the studied essential oils, thymol can be used as an environmentally friendly root-repellent agent instead of synthetic herbicides [31].

The aim of this study was to test the different *T. vulgaris* hydrolate solution (THS) concentrations against the seed germination and seedling lengths of some of the most important weed species that occur in Serbia: red-root amaranth (*Amaranthus retroflexus* L.), common lambsquarters (*Chenopodium album* L.), common purslane (*Portulaca oleracea* L.), cockspur grass (*Echinochloa crus-galli* (L.) P. Beauv.), Johnson grass (*Sorghum halepense* (L.) Pers.), and black nightshade (*Solanum nigrum* L.). The other aim was to study the phytotoxic potential of the hydrolate towards the most common field and vegetable crop seeds: soybean (*Glycine max* (L.) Merr.), sunflower (*Helianthus annuus* L.), maize (*Zea mays* L.), wheat (*Triticum aestivum* L.), carrot (*Daucus carota* subsp. *sativus* (Hoffm.) Schübl. & Martens), onion (*Allium cepa* L.), alfalfa (*Medicago sativa* L.), and white clover (*Trifolium repens* L.).

## 2. Results

### 2.1. Seed Germination

#### 2.1.1. Germination Percentage (GP)

Cultivated species: The highest germination percentage was registered in sunflower seeds (ranging from 73 to 96%), whereas the carrot seeds expressed the lowest germination percentage, ranging from 14 to 0% (Table 1).

Weed species: The tested hydrolate, depending on the hydrolate solution percentage, led to a reduction in germination ranging from 53 to 100%, with the exception of the black nightshade, in which 10 and 20% hydrolate solutions led to a reduction of ≤5%; by comparison, in the case of 50 and 100% THS, the reduction was 86 and 100%, respectively (Table 2).

#### 2.1.2. Coefficient of Velocity of Germination (CVG)

Cultivated species: The highest CVG values for the applied hydrolate solutions (from 10 to 100%) were noted in the following order: clover; soybean; soybean; sunflower (in the range from 25.70 to 38.00% day^−1^). The lowest CVG values for 10 and 20% THS were observed in carrot (11.76 and 12.50% day^−1^, respectively), whereas, for 50 and 100% hydrolate solutions, they were recorded for onion (17.95 and 13.51% day^−1^, respectively) (Table 1).

Weed species: The highest CVG value when 10% THS was applied was noted for common purslane seeds (22.77% day^−1^), whereas, in the case of 20 and 50% hydrolate solution, it was observed in cockspur (15.25 and 17.39% day^−1^, respectively). Conversely, Johnson grass expressed the lowest CVG values (in the range from 11.11 to 12.06% day^−1^) when 10, 20, and 50% hydrolate solutions were applied (Table 2).

#### 2.1.3. Germination Index (GI) and Germination Rate Index (GRI)

Cultivated species: Not taking into account the complete germination reduction, clover had the highest GI and GRI values for 10 and 20% hydrolate solutions (791/42.34 and 613/30.06% day^−1^, respectively), whereas sunflower expressed the highest values when 50 and 100% THS was applied (518/18.42 and 519/23.79% day^−1^, respectively). The lowest GI and GRI values for 10 and 20% hydrolate solutions were noted for carrot (35/1.70 and 3/0.12% day^−1^, respectively), whereas, in the case of 50 and 100% THS, the lowest values were observed for wheat (56/2.14% day^−1^) and onion (36/1.42% day^−1^) (Table 1).

Weed species: Not taking into account the complete germination reduction, the highest GI and GRI values were observed for common purslane (840/51.41 and 304/13.86% day^−1^, respectively), whereas the lowest values were noted for Johnson grass (in the range from 2/0.11 to 46/2.10% day^−1^) (Table 2).

#### 2.1.4. Median Germination Time (t_50_)

Cultivated species: Not taking into account the complete germination reduction, the highest t_50_ values for the applied hydrolate solutions (from 10 to 100%) were noted in the following order: carrot; carrot; maize; onion (in the range from 4.69 to 8.20 days); whereas the lowest values were observed as follows: sunflower; soybean; soybean; sunflower (ranging from 1.77 to 2.68 days) (Table 1).

Weed species: The highest t_50_ values were noted for Johnson grass (in the range from 7.90 to 8.50), whereas the lowest values were observed in the case of common purslane (10% hydrolate solution: –3.88) and cockspur (20 and 50%: 5.75 and 5.33) (Table 2).

The obtained results for the calculated germination indices (GP, CVG, GI, t_50_, and GRI) are shown in Table 1 and Table 2.

#### 2.1.5. Shoot and Root Length

The examination of the hydrolate effect on the initial growth of the studied field and vegetable seedlings showed that the sunflower seeds expressed a stimulative effect in terms of shoot and root seedling length when treated with the lower hydrolate concentrations (10%), whereas, in the case of the other tested plants, a statistically significant reduction, proportional to the increase in hydrolate concentration, was observed.

The TH reduced the seedling growth in all of the tested plant species, with the exception of black nightshade, which, like in the case of sunflower, exhibited shoot and root growth stimulation when treated with 10 and 20% hydrolate solution. However, 50% THS inhibited the growth of black nightshade seedlings (Table 3 and Table 4).

**Figure 1:**
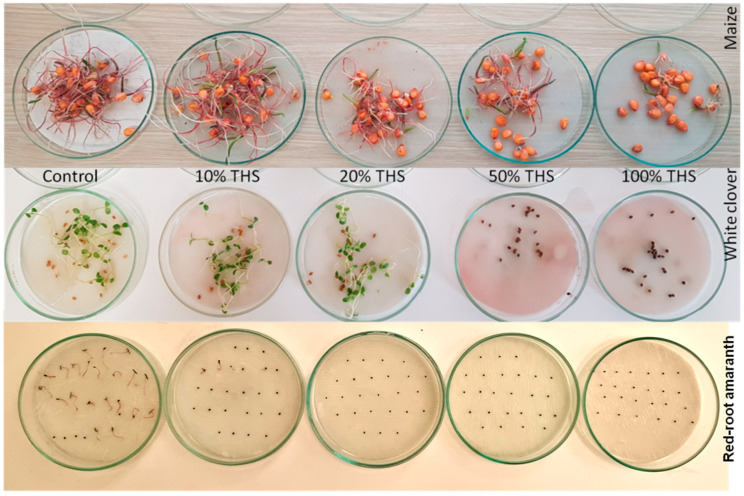
The effect of the applied *T. vulgaris* hydrolate solutions on maize and white clover.

The effect of the *T. vulgaris* hydrolate solutions on the germination rate and seedling length can be observed in Figure 1, where maize, white clover, and red-root amaranth are shown as the examples.

### 2.2. Biochemical Parameters

#### 2.2.1. MDA Content

The accumulation of the malondialdehyde (MDA) was notably higher in alfalfa seedlings after the treatment with 20% TH solution (115.74 nmol MDA/g FW). In the common lambsquarters seedlings, the amount of MDA after the treatment with 10% TH solution was 165.73 nmol MDA/g FW, whereas, in the case of the cockspur grass seedlings after treatment with the 20% hydrolate solution, it was 83.96 nmol MDA/g FW. The accumulation of the MDA indicates that 10 and 20% hydrolate extracts have a negative effect on these three species by inducing oxidative stress, along with the induction of the lipid peroxidation process. By comparison, the red-root amaranth seedlings showed higher oxidative stress in the control, whereas, in the case of Johnson grass, the highest MDA content was noted in the seedlings treated with 20% TH solution (74.51 nmol MDA/g FW). The results of the tested seedlings of the remaining species showed no statistically significant differences in terms of the lipid peroxidation intensity.

#### 2.2.2. SOD Activity

A significant decrease in SOD activity was detected in the case of maize (116.40 U/g FW) and soybean (5.69 U/g FW) seedlings when treated with the highest THS concentration, and in sunflower (33.85 U/g FW) and white clover (2.47 U/g FW) seedlings when treated with 20% THS. An increase in SOD activity was noted in the seedlings of wheat (all the tested concentrations, ranging between 122.96 and 125.39 U/g FW), carrot (10% hydrolate, 150.95 U/g FW), cockspur grass (20% hydrolate, 313.76 U/g FW), black nightshade (all the tested concentrations, with the highest in the case of 10% hydrolate, 236.92 U/g FW), and Johnson grass (all the tested concentrations, with the highest in the case of 20% hydrolate, 337.81 U/g FW). The alfalfa seedlings showed the highest SOD activity when treated with 20% hydrolate (282.83 U/g FW) and the lowest in the case of 10% hydrolate (175.61U/g FW). The results of the tested seedlings of the remaining species did not show any statistically significant differences in terms of the SOD activity (Table 5 and Table 6).

#### 2.2.3. O_2_.**^-^**/Radicals

Without taking into account common purslane, for which there was not enough plant material to carry out the analysis, the total quantity of removed superoxide anion radicals was proportionally higher with the increase in the TH concentrations in the seedlings of: wheat (319.63 nmol O_2_.**^-^**//g FW), onion (817.26 nmol O_2_.**^-^**//g FW), carrot (165.92 nmol O_2_.**^-^**//g FW), alfalfa (49.91 nmol O_2_.**^-^**//g FW), common lambsquarters (3366.46 nmol O_2_.**^-^**//g FW), and cockspur grass (536.88 nmol O_2_.**^-^**//g FW). The lowest quantities of removed superoxide anion radicals were noted in the seedlings of sunflower (56.08 nmol O_2_.**^-^**//g FW) and soybean (18.21 nmol O_2_.**^-^**//g FW) treated with 20% hydrolate, in the seedlings of white clover (25.45 nmol O_2_.**^-^**//g FW) treated with 10% hydrolate, and in those of maize (5.11 nmol O_2_.**^-^**//g FW) treated with 100% hydrolate. The results of the tested seedlings of the remaining species showed no statistically significant differences in terms of the quantity of removed superoxide anion radicals.

## 3. Discussion

The most important components of TH are the phenols carvacrol and thymol. Carvacrol and thymol have the potential to be used as bioherbicides and may help to reduce the use of synthetic herbicides and minimize damage to biodiversity and human health [32]. To date, these compounds have been associated with a phytotoxic effect on many weed and cultivated species. It was found that oregano (*Origanum acutidens* (Hand.-Mazz.) Ietsw.) essential oil and the phenols carvacrol and thymol had a phytotoxic effect on the seed germination and plant growth of red-root amaranth, common lambsquarters, and curly dock (*Rumex crispus* L.) [33]. The pepper-rosmarin (*Lippia sidoides* Cham.) essential oil, which contains thymol as its main component (84.90%), presented negative allelopathic effects on garden lettuce (*Lactuca sativa* L.) culture [34]. The phytotoxic activity of the *T. vulgaris* plant has been proven for various types of extracts and oils [35,36] and in soil experiments [37].

In this experiment, it was found that high concentrations of TH limited the seed germination of some weed species, such as common lambsquarters, red-root amaranth, and common purslane, whereas no negative effect was found on the germination of some field and vegetable crops. Seed germination efficiency depends on the seed’s size and weight; thus, large seeds contain more nutrients and are usually capable of faster germination and growth than small seeds [38]. Moreover, seedlings of large-seeded species have higher survival rates than small-seeded species [39]. This may partly explain the good germination of sunflower, soybean, and maize seeds, even after treatment with higher concentrations of the hydrolates, whereas, in small seeds of common lambsquarters, red-root amaranth, and common purslane, germination was completely absent after these treatments. In addition, germination of the mentioned weeds was significantly influenced by the concentrations of thymol and carvacrol. By investigating the sensitivity of common lambsquarters and red-root amaranth seeds to thymol and carvacrol, it was found that the germination was completely absent when 10 mg thymol and 9.8 mg carvacrol were applied [33]. Other studies concluded that ormadere (*Tanacetum chiliophyllum* var. *chiliophyllum* (Fisch. & Mey.) Sch. Bip.) essential oil, which is also rich in borneol, also inhibits the germination of these two weed species [40]. In a previous study [41], it was confirmed that carvacrol and thymol are the main compounds that induce the total inhibitory effect against seed germination of common purslane and cockspur grass. It was also found that carvacrol completely inhibits the germination of common purslane seeds [42]. According to [43], thymol has the greatest impact on cockspur grass, reducing its seed germination and shoot growth; this was confirmed in our research, in which 50 and 100% of TH rich in thymol completely inhibited the germination of this weed species, and 20% hydrolate reduced the seedlings’ length. In studies of the effect of oregano (*Origanum vulgare* L.) essential oil on Johnson grass, it was noted that carvacrol-rich (73.7%) essential oil inhibited Johnson grass seeds’ germination (52.7%) [44]. Our research confirms the hypothesis that carvacrol, in addition to thymol, plays a significant role in inhibiting the germination of this weed. Regarding black nightshade, a previous study proved that conehead thyme (*Thymbra capitata* (L.) Cav.) essential oil (which has carvacrol as its main compound) blocked black nightshade germination and seedling growth at 0.5 µL/mL [42]. Although our research showed that lower concentrations of TH stimulate germination and seedlings’ growth, higher concentrations (50 and 100%) led to their reduction.

Maize and sunflower showed the greatest resistance to the *T. vulgaris* hydrolates. The highest concentration of the applied hydrolate did not completely inhibit the germination of the maize seeds, although it was confirmed that thymol and carvacrol had an inhibitory effect on maize seeds’ germination [41] and growth [45].

According to [46], borneol, carvacrol, and thymol significantly inhibited the garden pepperwort (*Lepidium sativum* L.) radicle length by 10^−4^ M, and borneol reduced the garden radish (*Raphanus sativus* L.) radicle length by the same amount. When the effect of *T. vulgaris* essential oil on some crops and weeds was examined, it was found that cockspur grass inhibited the radicle and seedling length, whereas, in the case of garden radish, bell pepper (*Capsicum annuum* L.), and garden lettuce, seed germination was completely absent [47]. Treatment with the individual components (thymol, carvacrol, and borneol) showed that thymol stimulated the germination of garden radish seeds, but thymol and carvacrol completely inhibited the seed germination of bell pepper, garden lettuce, common lambsquarters, and common purslane. Moreover, thymol inhibited the cockspur grass radicle and seedling length. In our research, TH inhibited the germination and seedling growth of cockspur grass, common purslane, and common lambsquarters, and those of the other tested weed species, which is in accordance with the results of the above-mentioned studies.

Usually, the major components of hydrosol are the same as those present in the essential oils [3]. For the three main components of the studied THS (thymol, borneol, and carvacrol), the phytotoxic effects on some weed and cultivated species were determined.

Biochemical analysis showed that the tested crops expressed different sensitivity to THS, with alfalfa being the most sensitive. By comparison, a statistically significant increase in MDA accumulation was recorded in the tested weeds: common lambsquarters, cockspur grass, and Johnson grass. This means that the THS provoked stress in these plants and strongly affected the lipid peroxidation [48].

## 4. Materials and Methods

### 4.1. Tested Plants

In this experiment, the seeds of the field and vegetable crops, i.e., soybean, sunflower, maize, wheat, carrot, onion, alfalfa, and white clover, and of the weeds, i.e., red-root amaranth, common lambsquarters, common purslane, cockspur grass, Johnson grass, and black nightshade, were used. The field and vegetable crop seeds were obtained from The Institute of Field and Vegetable crops in Novi Sad, and the weed seeds were collected from several localities during 2019 and 2020, and confirmed and deposited at the Herbarium of The Department of Plant and Environmental Protection, Faculty of Agriculture, University of Novi Sad.

### 4.2. Hydrolate

**Figure 2:**
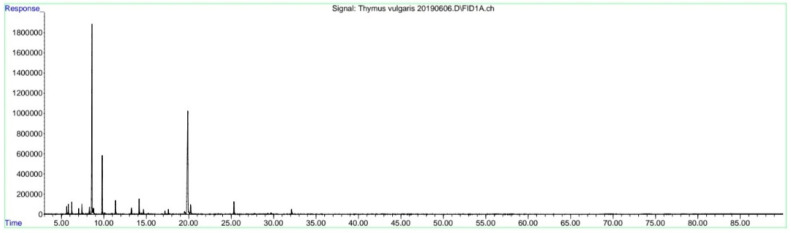
GC-FID chromatogram of *T. vulgaris* hydrolate.

*Thymus vulgaris* cv. “N19” plants were grown at The Institute of Field and Vegetable crops, Novi Sad (experimental field in Bački Petrovac). Steam distillation was performed in a small-scale distillation unit according to [49]. After two hours (according to the requirements of the European Pharmacopoeia), the essential oil was separated from the aqueous layer and the hydrolate was purified using filter paper and stored in the refrigerator at 8 °C during the whole experiment. Simultaneous distillation–extraction using the Likens–Nickerson apparatus was performed to isolate the volatile compounds, which were further analyzed by GC-FID and GC-MS, according to [50]. The main volatile compound in *T. vulgaris* hydrolate was thymol with 73.6%, followed by borneol (7.1%), carvacrol (4.4%), linalool (2.8%), terpinen-4-ol (2.8%), and 1-octen-3-ol (2.5%). The other 25 compounds were present in percentage shares of less than 1.0% (Figure 2).

The TH was diluted with distilled water in order to make 10, 20, 50, and 100% hydrolate solution, which was undertaken shortly before setting the experiment.

### 4.3. Seed Germination and Seedlings Length

After sterilization, which was performed according to [51], 25 seeds of each plant species were placed in a Petri dish (Ø 12 mm) on filter paper soaked with 10 mL of the particular hydrolate solution, i.e., distilled water in the case of the controls, in four replicates. The seeds of the tested weeds, and of the field and vegetable crops, were kept in a climate chamber at 22/20 °C during a 12 h photoperiod and at 60 ± 2% humidity for 10 days. The exceptions were Johnson grass and black nightshade, for which the seeds were kept at a higher temperature (30/26 °C). The seed germination rate and seedling length were recorded at the same time once a day during the whole experiment. The influence of TH on the tested seeds was determined by measuring the seedlings’ shoot and root length [52], and by recording the number of germinated seeds on the last day of the experiment.

In order to better understand the obtained results considering the germination of the tested seeds of the cultivated and weed species, several germination indices were calculated:

- Germination percentage (GP) [%] [53] represents the final germination percentage of the seed population and is calculated according to Equation (1):
  (1) $$\mathrm{GP}=\frac{\mathrm{Ng}}{\mathrm{Nt}}\times 10$$
  where Ng is the number of the germinated seeds and Nt is the total number of the seeds.

- Coefficient of velocity of germination (CVG) [% day^−1^] [54] represents the time required in order to reach the final germination percentage, and is calculated by Equation (2):
  (2) $$\mathrm{CVG}=\frac{\Sigma_{i=1}^{k}\;Ni}{\Sigma_{i=1}^{k}\;Ni\times Ti}\times 100$$
  where T*i* is the time from the start of the experiment to the *i*th interval; N*i* is the number of the seeds germinated in the *i*th interval (the number corresponding to the *i*th interval, not the accumulated number); and *k* is the total number of the intervals.

- Germination index (GI) [%] [55] reflects the germination speed; thus, a higher GI value indicates a faster germination rate. It is calculated according to Equation (3):
  (3) $$\mathrm{GI}=\left(10\times n_{1}\right)+\left(9\times n_{2}\right)+\cdots +\left(1\times n_{10}\right)$$
  where *n*_1_*, n*_2_…*n*_10_ represent the number of germinated seeds on the 1st, 2nd, and subsequent days until the 10th day; 10, 9…1 are the weights that are given to the number of germinated seeds on the 1st, 2nd, and subsequent days until the 10th day.

- Median germination time (t_50_) [time] [56] represents the time required in order to reach the 50% of the final germination and is calculated by Equation (4):
  (4) $$t_{50}=\frac{Ti+\left(\frac{N}{2}-Ni\right)\times \left(Tj-Ti\right)}{Nj-Ni}$$
  where N is the final number of germinated seeds; N*i* and N*j* are the total number of seeds germinated in adjacent counts at time T*i* and T*j*, when N*i* < $\frac{N}{2}$ < N*j*.

- Germination rate index (GRI) (% day^−1^) [57] (after a modification) reflects the germination speed without distinguishing between the days with higher or lower germination since the percentage is evenly spread across the time frame. It is calculated according to Equation (5):
  (5) $$\mathrm{GRI}=\frac{G_{1}}{1}+\frac{G_{2}}{2}+\cdots +\frac{G_{x}}{x}$$
  where G_1_ is the germination percentage × 100 on the 1st day after sowing; G_2_ is the germination percentage × 100 on the 2nd day after sowing, etc., and G*_x_* is the germination percentage × 100 on the *x*th day after sowing.

The seedlings of the tested plants were collected at the end of the experiment for the biochemical analysis.

### 4.4. Biochemical Analysis of the Tested Plants

For the determination of the biochemical parameters, 2 g of the fresh plant material (leaf) treated with TH, and the controls (untreated plants), were homogenized in phosphate buffer (10 mL; 0.1 M, pH 7.0). After centrifugation, the supernatants were used for the biochemical analyses. The biochemical parameters were determined spectrophotometrically using an UV/VIS spectrophotometer (Thermo Scientific Evolution 220 (Waltham, MA, USA)).

Superoxide dismutase (SOD) (EC 1.15.1.1) activity was determined according to the method of [58] with minor modifications. One unit of SOD activity was defined as the quantity of enzymes required to inhibit photochemical reduction of nitro blue tetrazolium (NBT) chloride by 50%. The SOD activity was expressed in U/g of the fresh weight (FW). The quantity of removed superoxide anion radicals was determined by the method of [59]. The total quantity of removed superoxide anion radicals (O_2_.**^-^**) is reported in nmol O_2_.**^-^** per g of the fresh weight (nmol O_2_.**^-^**/g FW). The content of malondialdehyde (MDA), which is the end product of the lipid peroxidation process, was measured at 532 nm using the thiobarbituric acid (TBA) test [58]. The total quantity of TBA-reactive substances is reported in nmol of the MDA equivalents per g of the fresh weight (nmol MDA/g FW).

### 4.5. Statistical Analysis

The values of the biochemical parameters are expressed as the mean ± standard error of the mean, and were tested by ANOVA followed by the comparison of the means by Duncan’s multiple range test (*p* < 0.05). The data were analyzed using TIBC STATISTICA version 14.

## 5. Conclusions

The studied THS showed an inhibitory effect on the weed species, i.e., red-root amaranth, common lambsquarters, common purslane, cockspur grass, Johnson grass, and black nightshade.

Regardless of the applied THS concentration, sunflower seeds showed the highest germination rates, whereas red-root amaranth seeds showed the highest sensitivity. The lower hydrolate concentrations (10%) had a stimulative effect in terms of sunflower shoot and root seedlings’ length, whereas, in the case of the other tested plants, a statistically significant reduction was noted.

The main compounds present in TH (thymol, borneol, and carvacrol) were able to inhibit the seed germination and seedling growth of several weeds, and exhibited a less phytotoxic effect on some of the tested field and vegetable crops. The tested hydrosol represents a potential source of an alternative and environmentally acceptable weed management compound for selective weed control.

## Figures and Tables

**Table 1:** The obtained results for the calculated germination indices in the case of the tested cultivated species treated with *T*. *vulgaris* hydrolate solutions.

Tested Plant	Concentration	GP (%)	CVG (% Day^−1^)	GI (−)	t_50_ (Time)	GRI (%/Day)
soybean	control	93	23.48	627	2.78	33.94
	10%	74	30.58	572	2.48	31.41
	20%	71	28.18	529	2.48	27.82
	50%	66	30.56	510	2.39	27.21
	100%	60	24.00	410	2.77	19.21
maize	control	97	22.35	633	4.15	27.45
	10%	80	19.66	473	4.80	19.36
	20%	82	18.34	455	4.56	18.13
	50%	65	18.36	361	4.69	15.81
	100%	21	23.33	141	3.25	8.71
onion	control	85	23.94	580	3.69	26.29
	10%	75	19.74	445	4.07	16.42
	20%	33	17.94	179	4.56	6.30
	50%	21	17.95	114	4.59	4.07
	100%	10	13.51	36	7.00	1.42
sunflower	control	89	33.21	711	1.81	44.89
	10%	96	34.78	780	1.77	49.72
	20%	88	19.43	515	4.18	18.13
	50%	83	21.01	518	3.81	18.42
	100%	73	25.70	519	2.68	23.79
alfalfa	control	83	35.78	681	2.17	36.08
	10%	85	32.69	675	2.18	35.73
	20%	76	23.03	506	2.76	24.99
clover	control	93	37.65	776	2.29	41.29
	10%	95	38.00	791	2.28	42.34
	20%	90	23.87	613	2.90	30.06
carrot	control	39	16.46	192	4.85	7.09
	10%	14	11.76	35	8.20	1.70
	20%	1	12.50	3	7.50	0.12
wheat	control	100	43.10	868	1.73	52.47
	10%	89	18.39	495	4.36	18.22
	20%	66	22.53	433	3.65	17.26
	50%	9	20.93	56	4.17	2.14

GP—germination percentage (ISTA, 2015); CVG—coefficient of velocity of germination (Jones and Sanders, 1987); GI—germination index (Bench et al., 1991); t_50_—median germination time (Farooq et al., 2005); GRI—germination rate index (Esechie, 1994 after modification).

**Table 2:** The obtained results for the calculated germination indices in the case of the tested weed species treated with *T*. *vulgaris* hydrolate solutions.

Tested Plant	Concentration	GP (%)	CVG (% Day^−1^)	GI (−)	t_50_ (Time)	GRI (%/Day)
red-root amaranth	control	83	24.78	578	2.56	30.41
	10%	26	14.86	111	6.29	4.03
cockspur	control	22	19.47	129	3.92	4.60
	10%	20	15.15	88	6.33	3.30
	20%	9	15.25	40	5.75	1.47
	50%	4	17.39	21	5.33	0.71
common purslane	control	100	38.46	840	1.88	51.41
	10%	46	22.77	304	3.88	13.86
Johnson grass	control	23	13.30	80	7.50	3.35
	10%	17	12.06	46	7.90	2.10
	20%	6	11.76	15	8.00	0.71
	50%	1	11.11	2	8.50	0.11
common lambsquarters	control	51	20.00	306	4.19	12.95
	10%	47	18.58	264	4.43	9.91
	20%	1	14.29	4	6.50	0.14
black nightshade	control	84	13.25	290	7.68	12.16
	10%	100	14.14	393	6.47	15.13
	20%	95	14.80	403	6.34	15.27
	50%	14	11.57	33	8.25	1.64

GP—germination percentage (ISTA, 2015); CVG—coefficient of velocity of germination (Jones and Sanders, 1987); GI—germination index (Bench et al., 1991); t_50_—median germination time (Farooq et al., 2005); GRI—germination rate index (Esechie, 1994 after modification).

**Table 3:** The shoot and root seedling length of the field and vegetable crop species treated with 10, 20, 50, and 100% *T. vulgaris* hydrolate solutions.

Tested Plant	Variable	Shoot Length (mm)	Root Length (mm)
onion	control	13.70 ± 10.15 ^a^	7.65 ± 6.40 ^a^
	10%	6.59 ± 5.69 ^b^	2.86 ± 2.80 ^b^
	20%	0.85 ± 1.98 ^c^	0.59 ± 1.44 ^c^
	50%	1.08 ± 1.64 ^c^	0.60 ± 0.98 ^c^
	100%	0.17 ± 0.53 ^c^	0.17 ± 0.53 ^c^
maize	control	25.26 ± 12.87 ^a^	69.29 ± 25.76 ^a^
	10%	19.49 ± 17.00 ^b^	54.31 ± 49.06 ^b^
	20%	13.80 ± 14.75 ^c^	29.66 ± 28.76 ^c^
	50%	10.78 ± 14.81 ^c^	20.09 ± 27.81 ^d^
	100%	4.87 ± 10.05 ^d^	5.53 ± 13.48 ^e^
sunflower	control	34.41 ± 21.64 ^a^	50.01 ± 38.16 ^b^
	10%	38.00 ± 19.68 ^a^	64.51 ± 46.27 ^a^
	20%	29.31 ± 23.66 ^b^	40.90 ± 41.06 ^b^
	50%	9.47 ± 7.91 ^c^	13.18 ± 10.70 ^c^
	100%	7.32 ± 6.54 ^c^	12.26 ± 11.13 ^c^
soybean	control	27.87 ± 13.03 ^a^	13.92 ± 8.65 ^b^
	10%	14.76 ± 11.18 ^b^	19.91 ± 19.83 ^a^
	20%	12.13 ± 9.80 ^b^	18.56 ± 18.65 ^a^
	50%	8.94 ± 7.37 ^c^	11.70 ± 12.40 ^b^
	100%	7.70 ± 7.41 ^c^	6.28 ± 6.61 ^c^
wheat	control	114.12 ± 19.17 ^a^	114.11 ± 19.14 ^a^
	10%	30.28 ± 26.61 ^b^	40.36 ± 34.36 ^b^
	20%	22.47 ± 26.56 ^c^	22.92 ± 26.09 ^c^
	50%	3.72 ± 13.82 ^d^	2.63 ± 9.69 ^d^
carrot	control	3.65 ± 7.38 ^a^	4.32 ± 6.95 ^a^
	10%	0.37 ± 1.07 ^b^	1.65 ± 4.54 ^b^
	20%	0.04 ± 0.40 ^b^	0.04 ± 0.40 ^c^
white clover	control	12.74 ± 4.99 ^a^	31.85 ± 16.72 ^a^
	10%	9.66 ± 3.47 ^b^	25.64 ± 10.86 ^b^
	20%	6.99 ± 3.26 ^c^	17.17 ± 10.18 ^c^
alfalfa	control	12.08 ± 7.71 ^a^	28.51 ± 19.45 ^a^
	10%	10.35 ± 5.35 ^b^	27.29 ± 17.64 ^a^
	20%	7.50 ± 5.44 ^c^	12.46 ± 11.92 ^b^

The data are mean values ± standard error. ^a–d^ Values without the same superscripts within each column differ significantly (*p* < 0.05).

**Table 4:** The shoot and root seedling length of the weed species treated with 10, 20, 50, and 100% *T. vulgaris* hydrolate solutions.

Tested Plant	Variable	Shoot Length (mm)	Root Length (mm)
common purslane	control	10.49 ± 2.55 ^a^	19.76 ± 4.53 ^a^
	10%	1.54 ± 1.78 ^b^	1.71 ± 2.01 ^b^
red-root amaranth	control	15.61 ± 8.32 ^a^	14.43 ± 7.72 ^a^
	10%	1.81 ± 3.29 ^b^	1.96 ± 3.41 ^b^
common lambsquarters	control	4.12 ± 5.63 ^a^	7.65 ± 12.77 ^a^
	10%	2.07 ± 3.21 ^b^	1.89 ± 2.46 ^b^
	20%	-	0.05 ± 0.50 ^c^
cockspur grass	control	8.90 ± 17.75 ^a^	10.70 ± 23.20 ^a^
	10%	6.13 ± 13.74 ^ab^	4.70 ± 11.07 ^bc^
	20%	3.08 ± 10.33 ^bc^	1.47 ± 5.88 ^b^
Johnson grass	control	10.48 ± 21.54 ^a^	10.38 ± 22.58 ^a^
	10%	2.38 ± 9.94 ^bc^	1.78 ± 7.33 ^bc^
	20%	5.52 ± 15.09 ^b^	4.81 ± 13.25 ^b^
	50%	0.42 ± 2.96 ^c^	-
black nightshade	control	2.57 ± 5.48 ^c^	9.69 ± 19.86 ^c^
	10%	10.96 ± 3.80 ^a^	43.88 ± 13.68 ^a^
	20%	8.77 ± 4.60 ^b^	19.64 ± 15.25 ^b^
	50%	1.22 ± 4.17 ^d^	2.97 ± 9.20 ^d^

The data are mean values ± standard error. ^a–d^ Values without the same superscripts within each column differ significantly (*p* < 0.05).

**Table 5:** Biochemical analysis of the treated field and vegetable crop species 14 days after the treatment.

Tested Plant	Variable	LP nmol/g FW	SOD U/g FW	nmol O_2_.^-^//g FW
wheat	control	69.40 ± 16.60 ^a^	119.09 ± 0.81 ^a^	58.01 ± 4.06 ^a^
	10%	67.91 ± 8.23 ^a^	124.46 ± 0.38 ^b^	89.84 ± 25.86 ^a^
	20%	48.09 ± 4.86 ^a^	122.96 ± 0.50 ^b^	252.08 ± 63.13 ^ab^
	50%	55.27 ± 8.72 ^a^	125.39 ± 1.31 ^b^	319.63 ± 103.44 ^b^
maize	control	62.00 ± 1.90 ^a^	120.44 ± 0.14 ^d^	15.83 ± 3.88 ^a^
	10%	38.66 ± 4.75 ^a^	118.91 ± 0.21 ^bc^	34.25 ± 9.08 ^bc^
	20%	69.25 ± 27.45 ^a^	119.53 ± 0.42 ^c^	19.66 ± 3.71 ^ab^
	50%	63.19 ± 23.16 ^a^	118.35 ± 0.07 ^b^	46.38 ± 1.21 ^c^
	100%	49.51 ± 8.59 ^a^	116.40 ± 0.00 ^a^	5.11 ± 0.63 ^a^
sunflower	control	52.20 ± 5.65 ^a^	82.66 ± 0.57 ^c^	57.63 ± 6.15 ^a^
	10%	42.03 ± 10.17 ^a^	50.60 ± 16.59 ^ab^	115.60 ± 7.12 ^b^
	20%	43.00 ± 6.69 ^a^	33.85 ± 11.59 ^a^	56.08 ± 16.80 ^a^
	50%	41.13 ± 4.83 ^a^	67.20 ± 5.48 ^bc^	76.26 ± 18.75 ^ab^
	100%	37.24 ± 6.66 ^a^	71.64 ± 0.26 ^bc^	99.39 ± 5.96 ^b^
soybean	control	95.20 ± 4.59 ^a^	107.96 ± 0.86 ^c^	22.38 ± 1.15 ^ab^
	10%	96.92 ± 8.93 ^a^	24.91 ± 0.99 ^b^	30.09 ± 1.71 ^bc^
	20%	97.15 ± 16.08 ^a^	23.01 ± 4.43 ^b^	18.21 ± 2.40 ^a^
	50%	86.53 ± 8.75 ^a^	10.68 ± 1.05 ^a^	33.75 ± 5.81 ^c^
	100%	89.89 ± 24.37 ^a^	5.69 ± 2.94 ^a^	19.71 ± 2.77 ^ab^
onion	control	36.87 ± 5.63 ^a^	111.58 ± 2.46 ^a^	90.44 ± 42.56 ^a^
	10%	37.92 ± 8.46 ^a^	97.41 ± 2.74 ^a^	290.53 ± 25.37 ^a^
	20%	35.17 ± 5.62 ^a^	116.34 ± 14.87 ^a^	817.26 ± 117.71 ^b^
carrot	control	18.03 ± 2.20 ^a^	99.19 ± 6.91 ^a^	60.83 ± 14.08 ^a^
	10%	18.54 ± 0.81 ^a^	150.95 ± 14.96 ^b^	165.92 ± 33.20 ^b^
white clover	control	43.73 ± 8.47 ^a^	23.46 ± 2.68 ^c^	46.48 ± 6.47 ^b^
	10%	38.65 ± 3.78 ^a^	8.72 ± 0.95 ^b^	25.45 ± 2.64 ^a^
	20%	60.90 ± 10.18 ^a^	2.47 ± 0.63 ^a^	30.07 ± 4.56 ^ab^
alfalfa	control	97.48 ± 20.25 ^ab^	188.94 ± 1.74 ^b^	17.77 ± 7.08 ^a^
	10%	51.35 ± 11.12 ^a^	175.61 ± 0.78 ^a^	24.64 ± 7.01 ^a^
	20%	115.74 ± 19.05 ^b^	282.83 ± 1.20 ^c^	49.91 ± 6.47 ^b^

The data are mean values ± standard error. ^a–d^ Values without the same superscripts within each column differ significantly (*p* < 0.05).

**Table 6:** Biochemical analysis of the treated weed species 14 days after the treatment.

Tested Plant	Variable	LP nmol/g FW	SOD U/g FW	nmol O_2_.^-^//g FW
common lambsquarters	control	18.48 ± 2.38 ^a^	79.52 ± 2.91 ^a^	154.49 ± 8.15 ^a^
	10%	165.73 ± 0.67 ^b^	334.59 ± 95.64 ^a^	3366.46 ± 412.48 ^b^
red-root amaranth	control	43.88 ± 2.81 ^b^	80.56 ± 3.06 ^a^	77.48 ± 1.87 ^a^
	10%	31.41 ± 2.42 ^a^	101.83 ± 10.51 ^a^	82.48 ± 19.03 ^a^
cockspur grass	control	37.81 ± 1.41 ^a^	142.56 ± 7.65 ^a^	151.52 ± 45.71 ^a^
	10%	32.90 ± 0.20 ^a^	126.47 ± 8.79 ^a^	256.70 ± 89.31 ^a^
	20%	83.96 ± 3.75 ^b^	313.76 ± 9.55 ^b^	536.88 ± 20.80 ^b^
black nightshade	control	48.92 ± 4.32 ^a^	84.67 ± 2.40 ^a^	305.66 ± 126.34 ^a^
	10%	48.46 ± 9.95 ^a^	236.92 ± 58.78 ^b^	1105.43 ± 9.45 ^a^
	20%	30.48 ± 2.91 ^a^	179.82 ± 3.76 ^ab^	644.68 ± 460.14 ^a^
	50%	35.23 ± 6.61 ^a^	100.55 ± 2.79 ^a^	420.61 ± 85.40 ^a^
Johnson grass	control	37.40 ± 1.77 ^ab^	77.59 ± 2.43 ^a^	224.38 ± 14.53 ^a^
	10%	33.36 ± 0.12 ^a^	166.76 ± 57.79 ^ab^	241.64 ± 46.46 ^a^
	20%	74.51 ± 2.82 ^c^	337.81 ± 31.68 ^c^	731.04 ± 290.72 ^a^
	50%	43.63 ± 2.98 ^b^	228.08 ± 3.93 ^b^	338.85 ± 8.69 ^a^

The data are mean values ± standard error. ^a–c^ Values without the same superscripts within each column differ significantly (*p* < 0.05).

## Data Availability

The data is contained within this manuscript. All data, tables and figures in this manuscript are original.
